# Cognitive training for freezing of gait in Parkinson’s disease: a randomized controlled trial

**DOI:** 10.1038/s41531-018-0052-6

**Published:** 2018-05-18

**Authors:** Courtney C. Walton, Loren Mowszowski, Moran Gilat, Julie M. Hall, Claire O’Callaghan, Alana J. Muller, Matthew Georgiades, Jennifer Y. Y. Szeto, Kaylena A. Ehgoetz Martens, James M. Shine, Sharon L. Naismith, Simon J. G. Lewis

**Affiliations:** 1 Parkinson’s Disease Research Clinic, Brain and Mind Centre, University of Sydney, Camperdown, NSW Australia; 2 Healthy Brain Ageing Program, Brain and Mind Centre & Charles Perkins Centre, University of Sydney, Sydney, NSW Australia; 3 School of Social Sciences and Psychology, Western Sydney University, Sydney, NSW Australia; 4 Department of Psychiatry and Behavioural and Clinical Neuroscience Institute, University of Cambridge, Cambridge, UK; 5 School of Psychology, Stanford University, Palo Alto, CA USA

## Abstract

The pathophysiological mechanism of freezing of gait (FoG) has been linked to executive dysfunction. Cognitive training (CT) is a non-pharmacological intervention which has been shown to improve executive functioning in Parkinson’s disease (PD). This study aimed to explore whether targeted CT can reduce the severity of FoG in PD. Patients with PD who self-reported FoG and were free from dementia were randomly allocated to receive either a CT intervention or an active control. Both groups were clinician-facilitated and conducted twice-weekly for seven weeks. The primary outcome was percentage of time spent frozen during a Timed Up and Go task, assessed both on and off dopaminergic medications. Secondary outcomes included multiple neuropsychological and psychosocial measures. A full analysis was first conducted on all participants randomized, followed by a sample of interest including only those who had objective FoG at baseline, and completed the intervention. Sixty-five patients were randomized into the study. The sample of interest included 20 in the CT group and 18 in the active control group. The primary outcome of percentage time spent frozen during a gait task was significantly improved in the CT group compared to active controls in the on-state. There were no differences in the off-state. Patients who received CT also demonstrated improved processing speed and reduced daytime sleepiness compared to those in the active control. The findings suggest that CT can reduce the severity of FoG in the on-state, however replication in a larger sample is required.

## Freezing of gait: a non-pharmacological approach

Cognitive training can reduce the severity of gait freezing in Parkinson’s disease (PD) patients on dopaminergic medication. The inability to move forwards despite the intention to walk is one of the most debilitating symptoms in patients with PD and has been linked to cognitive dysfunction. Simon Lewis at the University of Sydney, Australia, and colleagues examined the effects of cognitive training in patients with self-reported freezing of gait and without dementia. Patients receiving cognitive therapy twice a week for seven weeks showed a significant reduction in gait freezing compared to the control group. Interestingly, the effect was observed when patients had maximum benefit from their PD medication (on-state) but not in the off-state. Cognitive training also improved patients’ processing speed and reduced their daytime sleepiness highlighting the usefulness of non-pharmacological interventions in the treatment of PD.

## Introduction

Freezing of gait (FoG) is a disabling symptom of Parkinson’s Disease (PD), which presents as a “brief, episodic absence or marked reduction of forward progression of the feet, despite the intention to walk”.^1^ FoG is well-known to lead to falls^2^ and lower quality of life, making it an important target for treatment.^3^ The pathophysiological mechanism of FoG has been linked to executive dysfunction, particularly in aspects of cognitive control,^4^ which aligns with neuroimaging evidence showing fronto-parietal and fronto-striatal impairments.^5^ Recent meta-analytic data suggests that cognitive training (CT) is an effective^6^ and important^7^ behavioral intervention for improving cognition, and in particular executive functions, in patients with PD.

Given that these executive deficits have been hypothesized to underlie the pathophysiological mechanisms of FoG, it is plausible that reducing executive dysfunction via CT may lessen the severity of FoG, by mediating more effective fronto-striatal function.^8^ A number of studies have now shown that CT in PD can lead to neuroplastic changes by way of increased activity and functional connectivity in frontal-striatal regions.^9–11^ Given that FoG relates to dysfunction in these areas, it is reasonable to hypothesize that CT may facilitate more efficient processing between frontal and striatal regions, leading to a reduction of FoG severity. Interested readers are directed to a previous review from our group, which has provided more extensive evidence and rationale for this proposal.^12^

In this study, a double-blind randomized controlled trial was conducted to explore the efficacy of CT targeting executive functions in PD patients with FoG. We hypothesized that participants receiving CT would show improvements as illustrated by the reduced severity of FoG after completion of the intervention. Additionally, we anticipated that secondary outcomes including cognitive and psychosocial measures would show improvement following the CT program.

## Results

### Participants

**Fig. 1:**
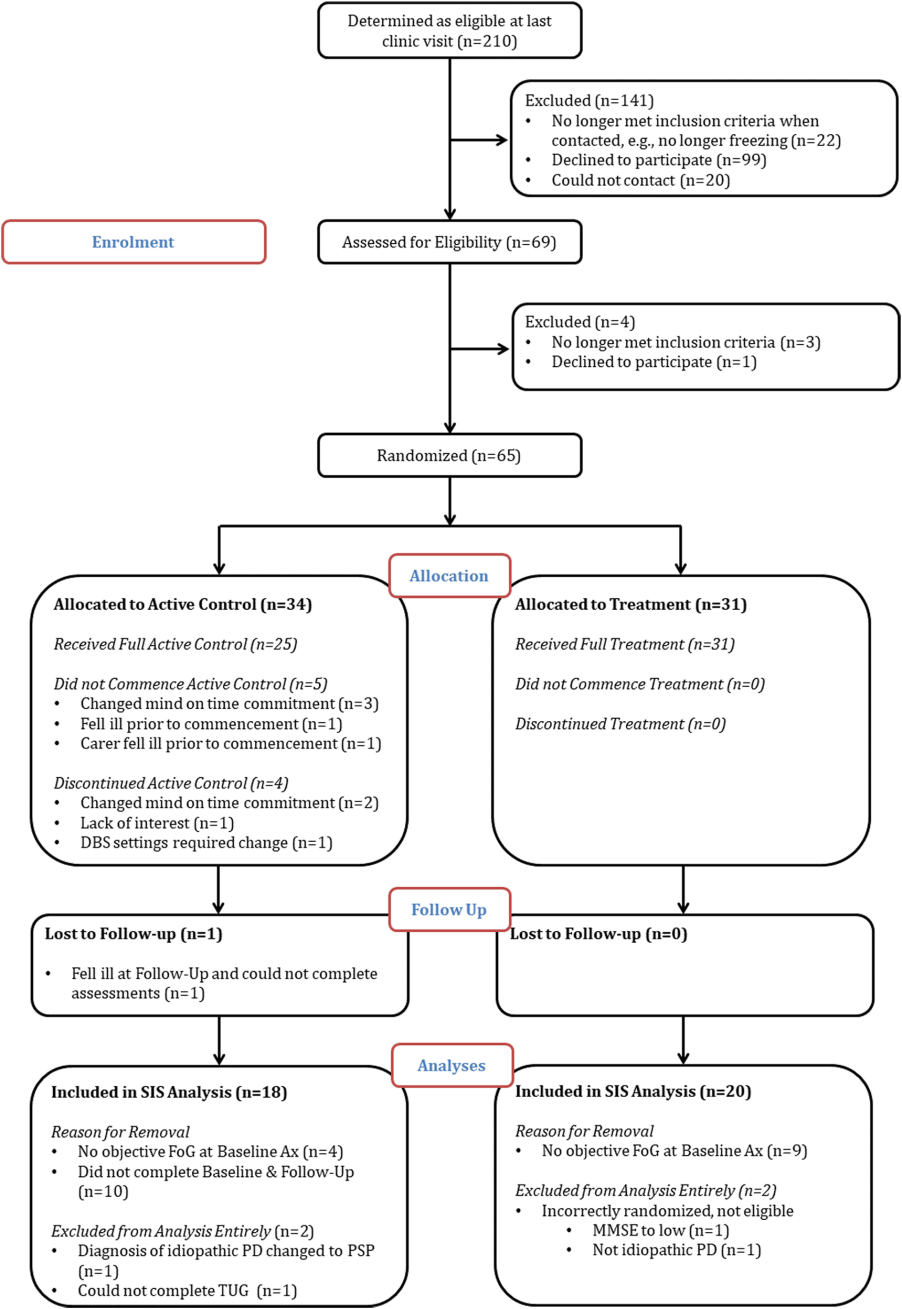
CONSORT Flow diagram

Figure 1 illustrates the flow of participants moving through the study. The first participant was randomized in April 2013 and the last in June 2015. There were nine dropouts in the active control condition (AC) (five prior to beginning the program) and one participant was lost to follow up. There were no dropouts in the CT group. In addition, in the AC group one participant was removed entirely from the analysis as their diagnosis was changed from PD to Progressive Supranuclear Palsy, and a second was removed as they could not complete the TUG assessments due to severe motor disability. Two participants were removed from the CT group due to inadvertent incorrect randomization. These participants were retrospectively identified as not meeting the original inclusion criteria and thus it was determined their data would not be analysed at any point.

**Fig. 2:**
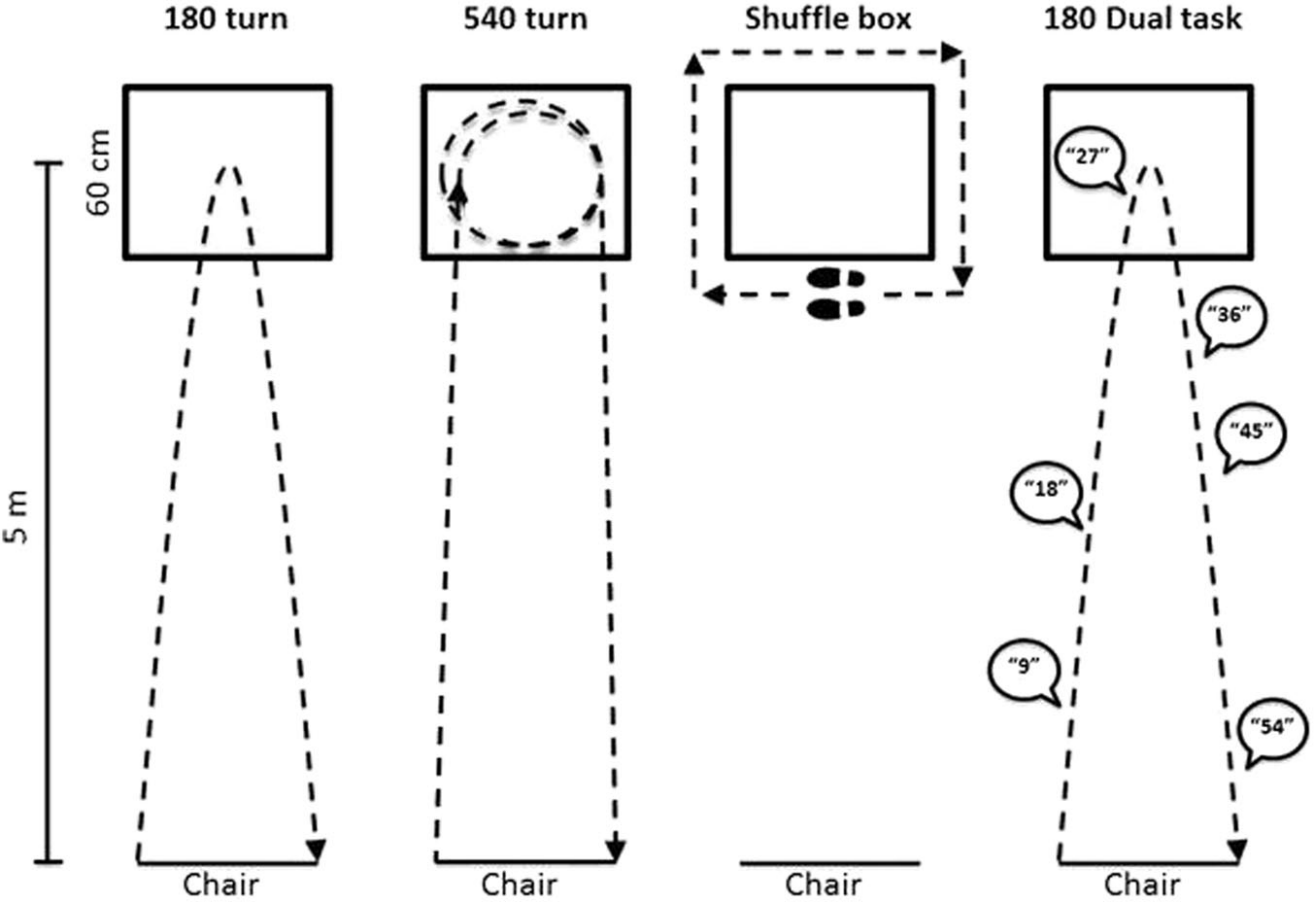
Each condition involved two trials with a left and right turn version. In the 180° condition, the participant walked to the box, turned around and returned to their chair; In the 540° condition, they completed a 540° turn in the box before returning to the chair; In the box condition, participants shuffled around the box, keeping their inside foot to the outside of the box; in the dual task condition, participants did the same as in the 180°, however completed a cognitive task as they walked. This was either naming the months backwards or multiples of 9 or 7 aloud. The %TF outcome was calculated by summing all FoG episodes across the four conditions, and dividing by the total time to complete across all conditions

Upon intervention completion, TUG scoring indicated that despite self-reporting FoG, nine participants from the CT and four from the AC group did not objectively exhibit FoG on baseline assessment. Therefore, we designed two analysis populations post-hoc. The full analysis set (FAS) included all participants randomized into the study, whether or not they dropped out, or showed objective FoG at BL. The sample of interest set (SIS) was decided upon as a post-hoc analysis, to account for the fact that a number of participants did not display FoG at baseline. The SIS therefore included only those participants who showed objective FoG on BL testing, as identified in the baseline TUG, and completed the study in full. The SIS population is considered the analysis of interest and is therefore what is reported in the results, however all analyses were initially run on the FAS sample to confirm no sampling bias. Demographic data for both the FAS and SIS samples are provided in Table 1.

**Table 1:** Demographic data of participants in both analysis samples

	FAS population		SIS population	
	AC group (*N* = 32)	CT group (*N* = 29)	AC group (*N* = 18)	CT group (*N* = 20)
Gender (M/F)	22/10	21/8	11/7	14/6
Age, years	68.50 (7.5)	68.48 (8.0)	69.61 (7.8)	69.70 (7.6)
Years since diagnosis	11.06 (6.6)	8.82 (4.9)	11.89 (6.6)	9.95 (4.4)
LEDD	934.71 (555.1)	769.37 (340.7)	975.43 (570.8)	828.8 (315.3)
Education, years	13.97 (3.2)	13.59 (3.2)	14.44 (3.5)	13.55 (3.4)
MMSE	28.16 (1.8)	27.72 (2.0)	28.56 (1.6)	27.35 (2.0)
Sessions attended	11.58 (3.7)	13.31 (0.9)	13.06 (1.2)	13.4 (0.9)
Days until follow up	8.91 (6.9)	6.48 (5.6)	9.11 (7.4)	7.20 (5.7)
Has DBS	6	4	3	4
MDS-UPDRS Motor on	36.16 (13.7)	36.83 (13.3)	33.44 (12.6)	38.70 (13.7)
MDS-UPDRS Motor off	43.76(11.7)	42.12 (12.4)	43.27 (11.8)	46.13 (10.9)
*Hoehn and Yahr stage*				
1	1	0	1	0
2	12	11	5	5
2.5	6	5	5	5
3	11	9	6	7
4	2	4	1	3

*LEDD* levadopa equivalence daily dose, *MMSE* mini-mental state examination, *DBS* deep brain stimulation, *MDS-UPDRS* Movement Disorder Society Unified Parkinson’s Disease Rating Scale

### Primary outcome

Results of the SIS analysis for the primary outcome are displayed in Table 2. This analysis showed that patients in the CT group showed a large and statistically significant reduction in FoG severity in the on-state compared to participants in the AC. There was no difference in the off*-*state. The FAS analysis was consistent with these results, suggesting that the SIS sample was not biased. We did not compare performance across each of the four conditions separately, on and off as this was not part of the predetermined outcome plan, and secondly, we felt it was an inappropriate exploratory analysis given the smaller than anticipated sample.

**Table 2:** Primary outcome data between groups before and after intervention

	AC group	CT group	Comparison of change
*%TF on* ^a^			
BL	6.61 (3.92, 11.15)	9.16 (5.52, 15.19)	0.3 (0.14, 0.62); T = −3.36; *p* = 0.002; *d* = 1.02
FU	11.99 (7.11, 20.23)	4.95 (2.99, 8.21)	
Change from BL	1.81 (1.08, 3.05)	0.54 (0.32, 0.90)	
*%TF off* ^a^			
BL	16.61 (9.22, 29.93)	8.02 (4.59, 13.98)	1.07 (0.64, 1.76); T = 0.26; *p* = 0.800; *d* = 0.08
FU	15.44 (8.57, 27.81)	7.94 (4.55, 13.85)	
Change from BL	0.93 (0.64, 1.34)	0.99 (0.70, 1.40)	

^a^Analysis conducted on Log_10_ transformed data and represented by geometric means. The change from baseline is a ratio of the geometric means at follow-up compared with baseline. The comparison of the changes from baseline is the ratio of the change from baseline for the CT group compared the AC group. 95% CIs are presented in brackets. Direction of change and statistical significance was matched in the FAS analysis

### Secondary outcomes

Results of the SIS analysis for the secondary outcomes are displayed in Table 3. In the SIS analysis, there were no statistically significant differences for any of the secondary outcomes over time between groups.

**Table 3:** Secondary outcome data between groups before and after intervention

	AC group	CT group	Comparison of change
*MOCA*			
BL	25.33 (23.57, 27.09)	24.15 (22.48, 25.82)	0.84 (−1.38, 3.06); T = 0.77;
FU	25.39 (23.63, 27.15)	25.05 (23.38, 26.72)	*p* = 0.45;
Change from BL	0.06 (−1.55, 1.67)	0.9 (−0.63, 2.43)	*d* = 0.24
*HVLT-R total*			
BL	19.39 (16.83, 21.95)	18.50 (16.07, 20.93)	−1.93 (−4.75, 0.88);
FU	21.72 (19.16, 24.28)	18.90 (16.47, 21.33)	T = −1.39; *p* = 0.17;
Change from BL	2.33 (0.29, 4.38)	0.40 (−1.54, 2.34)	*d* = 0.44
*HVLT-R delayed*			
BL	5.67 (4.23, 7.10)	5.00 (3.64, 6.36)	−0.53 (−2.21, 1.15);
FU	6.35 (4.89, 7.80)	5.15 (4.49, 6.751)	T = −0.64; *p* = 0.53;
Change from BL	0.68 (−0.55, 1.92)	0.15 (−0.99, 1.29)	*d* = 0.19
*Digit span total*			
BL	16.00 (14.41, 17.59)	14.85 (13.34, 16.36)	−0.93 (−2.18, 0.32);
FU	16.78 (15.19, 18.37)	14.70 (13.19, 16.21)	T = −1.50; *p* = 0.14;
Change from BL	0.78 (−0.13, 1.69)	−0.15 (−1.01, 0.71)	*d* = 0.47
*TMT-A time* ^a,b^			
BL	44.74 (35.40, 56.48)	50.31 (40.10, 63.05)	0.78 (0.64, 0.95);
FU	45.67 (36.14, 57.64)	39.77 (31.65, 49.90)	T = −2.57; *p* = 0.01;
Change from BL	1.02 (0.89, 1.18)	0.79 (0.69, 0.91)	*d* = 0.62
*TMT-B time* ^a^			
BL	119.11 (95.63, 148.31)	136.08 (110.52, 167.51)	0.88 (0.75, 1.04);
FU	120.96 (97.11, 150.61)	122 (99.12, 150.29)	T = −1.50; *p* = 0.14;
Change from BL	1.02 (0.90, 1.15)	0.90 (0.80, 1.01)	*d* = 0.48
*VF-phonemic*			
BL	37.22 (32.26, 42.19)	31.55 (26.84, 36.26)	−0.47 (−3.96, 3.03);
FU	37.89 (32.93, 42.85)	31.75 (27.04, 36.46)	T = −0.27; *p* = 0.79;
Change from BL	0.67 (−1.87, 3.2)	0.20 (−2.21, 2.61)	*d* = 0.09
*VF-semantic*			
BL	33.22 (28.46, 37.99)	30.45 (25.93, 34.72)	−1.03 (−5.28, 3.23);
FU	34.00 (29.23, 38.77)	30.20 (25.68, 34.72)	T = −0.49; *p* = 0.63;
Change from BL	0.78 (−2.31, 3.87)	−0.25 (−3.18, 2.68)	*d* = 0.15
*VF-switching total*			
BL	12.11 (10.54, 13.68)	9.70 (8.21, 11.19)	1.18 (−0.51, 2.87);
FU	11.78 (10.20, 13.35)	10.55 (9.06, 12.04)	T = 1.42; *p* = 0.16;
Change from BL	−0.33 (−1.56, 0.89)	0.85 (−0.31, 2.01)	*d* = 0.45
*VF-switching accuracy*			
BL	10.83 (9.04, 12.63)	8.90 (7.20, 10.60)	−0.09 (−2.54, 2.35);
FU	10.28 (8.48, 12.07)	8.25 (6.55, 9.95)	T = 0.08; *p* = 0.94;
Change from BL	−0.56 (−2.33, 1.22)	−0.65 (−2.33, 1.03)	*d* = 0.02
*SDMT total*			
BL	35.44 (30.00, 40.89)	33.95 (28.78, 39.12)	0.47 (−3.74, 4.68);
FU	37.22 (31.77, 42.67)	36.20 (31.03, 41.37)	T = 0.23; *p* = 0.82;
Change from BL	1.78 (−1.28, 4.83)	2.25 (−0.65, 5.15)	*d* = 0.07
*AGN latency*			
BL	595.75 (547.92, 643.58)	657.65 (608.42, 706.89)	−29.89 (−66.37, 6.58);
FU	586.42 (538.89, 633.95)	618.43 (569.19, 667.67)	T = −1.67; *p* = 0.10;
Change from BL	−9.33 (−34.34, 15.68)	−39.22 (−65.78, −12.67)	*d* = 0.62
*AGN omissions* ^a^			
BL	2.85 (1.49, 4.94)	4.35 (2.42, 7.37)	0.70 (0.46, 1.06);
FU	4.26 (2.42, 7.08)	4.15 (2.29, 7.05)	T = −1.73; *p* = 0.09;
Change from BL	1.37 (1.03, 1.82)	0.96 (0.71, 1.3)	*d* = 0.61
*AGN commissions* ^a^			
BL	5.05 (3.35, 7.41)	4.28 (2.76, 6.42)	0.83 (0.54, 1.3);
FU	5.15 (3.45, 7.51)	3.48 (2.19, 5.30)	T = −0.83; *p* = 0.41;
Change from BL	1.02 (0.75, 1.38)	0.85 (0.62, 1.17)	*d* = 0.26
*HADS total*			
BL	10.78 (7.85, 13.71)	12.70 (9.92, 15.48)	−1.98 (−5.38, 1.42);
FU	11.56 (8.63, 14.48)	11.50 (8.72, 14.28)	T = −1.18; *p* = 0.25;
Change from BL	0.78 (−1.69, 3.24)	−1.2 (−3.54, 1.14)	*d* = 0.37
*PDQ-39 total*			
BL	26.69 (20.81, 32.58)	28.21 (22.63, 33.79)	−3.33 (−8.07, 1.41);
FU	27.32 (21.43, 33.20)	25.50 (19.92, 31.09)	T = −1.42; *p* = 0.16;
Change from BL	0.62 (−2.82, 4.06)	−2.71 (−5.97, 0.55)	*d* = 0.45
*CBI total*			
BL	18.74 (10.62, 26.86)	33.51 (26.21, 40.81)	−4.51 (−10.66, 1.64);
FU	20.85 (12.66, 29.04)	31.11 (23.86, 38.36)	T = −1.49; *p* = 0.15;
Change from BL	2.11 (−2.52, 6.73)	−2.4 (−6.45, 1.65)	*d* = 0.53
*SCOPA-S day* ^b^			
BL	4.77 (3.16, 6.38)	6.31 (4.72, 7.89)	−1.66 (−3.27, −0.05);
FU	5.00 (3.39, 6.62)	4.88 (3.32, 6.44)	T = −2.10; *p* = 0.04;
Change from BL	0.24 (−0.91, 1.38)	−1.43 (−2.56, −0.29)	*d* = 0.56
*SCOPA-S night*			
BL	5.33 (3.18, 7.48)	6.00 (3.94, 8.06)	−0.24 (−2.17, 1.68);
FU	5.61 (3.46, 7.76)	6.03 (3.97, 8.09)	T = −0.26; *p* = 0.80;
Change from BL	0.28 (−1.09, 1.64)	0.03 (−1.32, 1.39)	*d* = 0.06

*MOCA* Montreal cognitive assessment, *HVLT-R* Hopkins verbal learning test-revised, *WMS-III* Wechsler Memory Scale, *VF* verbal fluency, *DKEFS* Delis–Kaplan executive function system, *SDMT* symbol digit modalities test, *TMT* trail making test, *AGN* affective go-no-go test, *HADS* Hospital Anxiety & Depression Scale, *SCOPA* Scales for Outcomes in Parkinson’s disease, *PDQ-39* the Parkinson’s disease questionnaire, *CBI-R* Cambridge behavioral inventory-revised

^a^Analysis conducted on Log_10_ transformed data and represented by geometric means. The change from

baseline is a ratio of the geometric means at follow-up compared with baseline. The comparison of the changes from baseline is the ratio of the change from baseline for the CT group compared the AC group

^b^Results are adjusted for DDE as a covariate. 95% CIs are presented in brackets

### Covariate analysis

The covariate analysis showed that the results for the primary outcome remained unchanged by the introduction of covariates (i.e., still statistically significant). However, in terms of secondary outcomes, the inclusion of DDE as a covariate led to TMT-A and daytime sleep disturbance scores becoming significant, with those in the CT group improving compared to the AC.

## Discussion

This pilot study represents one of the largest RCTs of CT to date in PD. Though interpretation of the results must remain cautious owing to the limitations outlined below, the results allude to the potential for CT to reduce the severity of FoG in people with PD. We showed that CT led to a large and significant reduction of FoG severity compared to AC while in the on*-*state, but this was not replicated in the off-state. These results were consistent, whether we included participants who did not display FoG at baseline or not, and when accounting for covariates. We suggest these results warrant larger scale replication, employing the suggested methodological adjustments we provide below.

The result of FoG only improving during the on-state is noteworthy. Firstly, we preface this discussion by stating this is the clinically relevant behavioral state, as patients in their day-to-day life would take dopaminergic medications as prescribed to minimize time in the off*-*state. Our *provisional* supposition to explain this result is that participants in the off-state were too impaired to benefit from any of the potential changes initiated through CT. Training was expected to impact frontal processing and also occurred in the on*-*state. In the dopamine depleted state, it is conceivable that the striatal dysfunction overshadowed any benefit of CT,^8,13^ and FoG could not be improved. Our future analysis of functional neuroimaging outcomes in this study may be able to unravel this further.

A number of trials in older adults have now shown that CT can have a beneficial impact on multiple gait parameters.^14–16^ In PD specifically, a pilot study by Milman and colleagues^17^ showed that 12 weeks of CT could improve TUG performance. Unfortunately, this pilot study did not employ a control group. Therefore, the current results are an important extension showing improvements on TUG performance via the reduction of FoG, compared to an active control group.

Given that FoG was reduced in the CT group, we expected there to be additional improvements in tests of EF, which were presumed to underlie any improvement of FoG. However, changes on these outcomes did not reach statistical significance. We were therefore unable to confirm the hypothesis that improving EF would be the driver of reduced severity of FoG. It is possible that our smaller sample size was a factor however (though it was deemed inappropriate to conduct post-hoc power analysis^18^). Indeed, there were near medium-sized effect sizes (*d* ≥ 0.45) for many of the executive tests we anticipated improvements on including TMT-B, and shift-measures of the affective go-no-go test (AGN) and verbal fluency (VF) (see Table 2). We do note however that when adjusting for the effect of dopaminergic medication, the CT group did show medium-sized significant improvements compared to AC in processing speed and daytime sleepiness.

### Limitations

This study has limitations which warrant consideration. The first is that we did not meet the projected sample size target due to feasibility issues with recruitment. In addition, there were a number of dropouts in the AC group, although we note that over half of these dropouts occurred prior to commencement of the intervention and only one was due a lack of interest. The third issue was that participants were randomized prior to TUG scoring. This was necessary to avoid delay to participants being enrolled as TUG scoring is time consuming, requiring skilled and trained raters. Nonetheless, we attempted to address these limitations by running the FAS analysis, which confirmed our primary result.

Additionally, despite random allocation, the groups were unbalanced in their baseline FoG severity. The CT group had more FoG than the AC in the on and less in the off*-*state. We stratified the randomization by cognitive functioning (MOCA scores), however it may be more appropriate in future to stratify by objective FoG scores at baseline. We highlight however that the results remained when accounting for the impact of LEDD, and that the on-state is the clinically meaningful state. Related to this unbalanced severity, it is important to highlight that the AC group actually had worse FoG at follow-up compared to the CT group in the on-state and this pattern of results was replicated in the FAS analysis. Replication with a larger sample is needed to demonstrate if this is a reliable finding, or represents the variability found in small samples such as this one.

### Future directions

We believe there is reason to be hopeful for the use of these trials in the future. Feedback from participants and family members involved in the groups was overwhelmingly positive, our pilot results highlight positive trends, and the importance of nonpharmacological trials including CT has become increasingly clear.^7,19,20^ We suggest that replication of this trial is warranted. However, with the hope of improving any future work learning from some of the issues that were raised during this study, we suggest authors consider some of the following suggestions.

Future studies where possible should aim to score FoG severity prior to enrollment. A certain threshold for severity (e.g., >5%) should be specified for eligibility, and stratification across groups could also be based on this. Where possible, additional methods of FoG measurement could increase the reliability of %FoG scores. This could be done through measures such as gait mats and accelerometry data, and also repeat TuG assessments to address measurement variability. Multisite recruitment would increase the potential for sample size without relying on home-based CT, which we do not believe would be a viable option,^21^ particularly in this sample. The inclusion of additional data to aid analysis such as measurement of expectancy effects and CT training data can be useful. Finally, we did not include a long-term follow up assessment. This has often been used as a criticism against CT, though we rebut that very few interventions elicit sustained improvements after the cessation of treatment. Thus, it is likely that clinically, CT needs to be continuously delivered in order to continue any found benefits, just like most other interventions (e.g., exercise, medications etc). Nevertheless, obtaining a better understanding of how long such results are maintained^22^ is useful for future trial design and clinical applications, and thus future studies could try to obtain this information if feasible.

## Conclusions

The current study provides preliminary evidence that CT can reduce the severity of FoG in PD during the on-state. This improvement was seen without concurrent, significant changes to executive functioning (despite near-medium sized effects on these measures), but in the context of improved processing speed and daytime sleep disturbance. Despite the limitations of this study, these results add to the growing body of evidence showing that CT is a useful therapeutic technique worthy of continued exploration in PD.

## Methods

### Study registration

This study was registered in 2013 on the 5 April through the Australian and New Zealand Clinical Trials Registry (ACTRN12613000359730) and was approved by the Human Research Ethics Committee of the University of Sydney. Written informed consent was obtained from all participants.

### Eligibility

Eligible participants were those diagnosed with idiopathic Parkinson’s disease based on the UK Brain Bank clinical criteria,^23^ with self-reported FoG at the time of assessment, and who were free from dementia as determined by a score of ≥24 on the mini-mental state examination (MMSE).^24^

### Recruitment

The study was advertised in a local PD community magazine as well as local PD community support groups. Potential participants were also recruited from the Parkinson’s Disease Research Clinic at the Brain and Mind Centre, University of Sydney. Interested participants were invited to participate if they had previously reported a positive score on Question 3 of the Freezing of Gait Questionnaire (FOG-Q): “do you feel that your feet get glued to the floor while walking, making a turn, or trying to initiate walking (freezing)?”.^25^

Prior to recruitment, we used baseline data from a previously published trial^26^ to conduct a power analysis using a conservative effect size estimate of at least 0.2 in the study’s primary outcome. This suggested the minimum sample size required for each group was 39 (based on power = 0.80 and *α* = 0.05).

### Study design

The study was a double-blind randomized active controlled trial. Interested patients were enrolled by CCW and LM after they met eligibility criteria during a baseline assessment and were then randomized into either the CT or an AC group. Conditions were masked as either “morning” or “afternoon” sessions, and the order of these was randomized between recruitment waves prior to trial commencement. In order to facilitate blinding, participants were told that each session involved different computerized activities, but were not explicitly told of a treatment or control group. Randomization of participants and morning/afternoon sessions was carried out using a randomly generated number sequence allocated by a blinded researcher not involved in trial recruitment, data gathering, assessments or training. Randomization was undertaken using permuted blocks and stratified by cognitive functioning, with strata defined by Montreal cognitive assessment (MOCA) scores of <26 or ≥26. Participants were advised of their allocation into the morning or afternoon session by way of sealed opaque envelopes delivered by CCW upon completion of the baseline assessments. Post-intervention assessments were conducted by clinicians who were blinded to treatment allocation. All participants allocated to the AC group were offered the opportunity to complete CT after their involvement in the trial was complete. Ten participants elected to complete this, with those who declined citing time commitments as the primary reason.

### Assessments

Baseline and post-intervention assessments were each completed in two parts: on and off-medications. The on*-*state assessment included a neuropsychological test battery, psychosocial measures, part III of the Movement Disorders Society’s revision of the Unified Parkinson’s Disease Research Scale (MDS-UPDRS),^27^ and a modified timed up-and-go (TUG) task. These assessments were completed in a random counterbalanced order and took approximately 2.5 h to complete. Baseline assessments were conducted within 3 weeks prior to training commencement, and follow-up assessment was within 3 weeks of the intervention finishing. The practically defined off*-*state assessment was completed in the morning on a different day when participants were asked not to take their usual Parkinson’s medication until after the assessment, and comprised a repeated MDS-UPDRS part III and TUG, taking approximately 1 h. Those with deep brain stimulation did not complete the off-state assessment. A random subset of participants also underwent neuroimaging, however the investigation of any training-induced changes are a tertiary outcome and are therefore not included in the current manuscript.

### Primary outcome

The primary outcome was percentage of time spent frozen (%TF) across all four trials of a TUG assessment. Percentage was chosen as the primary outcome as it was anticipated to be more sensitive than a reduction on the FOG-Q, whilst accounting for inter-individual variability in gait speed and the variable duration of freezing episodes across TUG tasks.^26^ In each task, the participant was requested to get up from a chair, walk to a square box shape taped to the floor five meters ahead and complete both a left and a right turn (see Fig. 2). TUGs were video recorded and then scored independently post-assessment. Six scorers (MG, JMH, AJM, MG, JYYS, and KAEM) were randomly distributed videos of the TUGs. Scorers were given baseline and follow-up TUG videos in a random order for the same participant, to minimize pre-post scoring variability. FoG was tagged in the video at any point when a participant made a paroxysmal and involuntary cessation of normal progression of the feet through the task. This included a typical trembling of the feet, short shuffling steps of a few centimetres in length or a complete motor block.^28^

The %TF outcome was calculated by summing all FoG episodes across the four conditions, and dividing by the total time to complete across all conditions. Inter-rater variability amongst blinded scorers was strong, and calculated by all scorers being given a random selection of the same six videos to independently score. The intraclass correlation coefficient was .902 across all FoG episodes (average %TF: 9.56%). We note that two of the six videos by chance did not contain FoG, however they were included to confirm no false-positive scoring had occurred. As this inflated reliability across scorers however, we re-calculated the coefficient with the two videos removed to be sure. Scoring was still accurate across raters (.865) (average %TF: 14.31%).

### Secondary outcomes

#### Cognitive assessment

To assess global functioning for descriptive purposes, the MMSE^24^ and MOCA^29^ were used. Total and delayed Hopkins verbal learning test-revised scores were used to assess verbal memory.^30^ To assess attention and working memory, the total score from the Digit Span subtest of the Wechsler Memory Scale was used.^31^ To assess verbal fluency, the total words generated on each condition of the VF subtest of the Delis–Kaplan executive function system was used.^32^ In this test, part 1 measures phonemic fluency across three letters, part 2 measures semantic fluency across two categories while parts 3 and 4 assess switching between two differing categories. Part 3 represents the total number of correct items, while part 4 represents the total number of correct switches. Processing speed was assessed by the number of correct responses on the oral symbol digit modalities test.^33^ For these measures, higher scores are indicative of better performance.

To assess processing speed and cognitive flexibility, times for parts A and B respectively of the trail making test (TMT) were used.^34^ Finally, the AGN of the Cambridge neuropsychological test automated battery^35^ was used as a computerized measure of inhibitory control/switching. Mean latency post-switch was used to determine performance in addition to the number of commissions or omissions. For these tests, lower scores were indicative of better performance. Alternate versions of each test with the exception of Digit Span (not available) were used at baseline and follow-up to minimise potential practice effects.

#### Psychosocial measures

Participants completed several questionnaires targeting mood and wellbeing. The Hospital Anxiety & Depression Scale was used to assess anxious and depressive symptoms.^36^ The Scales of Outcomes in PD (SCOPA)-Sleep was used to assess sleep quality in terms of both daytime sleepiness and night time sleep disturbance.^37^ The Parkinson’s Disease Questionnaire (PDQ-39)^38^ was used as a measure of quality of life. Finally, if a participant lived with a carer, the Cambridge behavioral inventory-revised^39^ was used as an informant report of cognitive and behavioral changes. For all of these scores, a higher score was indicative of more substantial impairment.

#### Intervention

Both the CT and AC groups attended sessions at the Brain and Mind Centre at the University of Sydney in our designated CT laboratory, and both conditions were matched in terms of time, clinician contact, computer use and social interaction. In accordance with our previous CT programs for older adults (see,^40,41^) the intervention was completed in a group format (*n* ≤ 10), and comprised of 2-h sessions, twice weekly over 7 weeks (14 sessions in total). Both groups were supervised and facilitated by CCW & LM. The first hour of the session was identical across CT and AC groups: (i) 30–45 min was designated to psycho-education on a number of topics relevant to PD including cognition, sleep and mood, and was delivered by multidisciplinary specialists and researchers from the Brain and Mind Centre; (ii) participants then took an enforced break of 10–15 min. This first hour, whilst not CT per-se, was included for both conditions as a means of increasing participant engagement and has previously been shown to support our excellent program adherence rates.^40,41^ The second hour of the session differed across CT and AC groups:

(A) CT: Participants in this group completed a program of computerized CT tasks, selected for their focus on executive functions and on the basis of our previous experience employing the “Neuropsychological Educational Approach to Remediation” approach^41,42^ in providing computerized CT to >400 older adults,^41,43,44^ including those with PD.^40^ Tasks included designated “brain training” programs (e.g., Attention Process Training-III^45^) as well as commercially-available software (e.g., computerised Sudoku), which were determined by clinical neuropsychologists (LM, SLN) to target the cognitive processes of most interest to FoG (inhibitory control, attentional set-shifting, working memory, processing speed and visuospatial skills).^4,12^ Performance was monitored by the facilitators, with the focus of progressively making the tasks more difficult where possible for the participant. These changes were done in an individualized manner based on performance and in consultation with the participant. Therefore while the tasks delegated for each session where standardized across all participants, there were differences in how far each progressed in terms of difficulty. The majority of exercises provided the participant with feedback in the form of scores, and this was further discussed between facilitators and participants to help them better understand their performance.

(B) AC: Participants in this group completed a series of non-specific computer-based tasks including predominantly watching informative nature videos and answering content-related questions as previously used,^46^ as well as online “treasure hunts” devised by our team. These tasks were designed to provide broader, generalized cognitive engagement compared to the targeted focus on specific cognitive functions in the CT condition. Therefore, those in this group were not expected to have reduced FoG severity but were rather intended to match for clinician and peer contact, along with computer use.

#### Statistical analysis

To minimize any potential bias, statistical analyses were conducted by a consultant statistician experienced in RCTs who was based at the University of Sydney (see acknowledgements) and who was not involved in any other aspect of the trial. Data was analysed using SAS software version 9.4.

The analysis took the form of a mixed effects model using fixed effects fitted to all endpoints, to test the null hypothesis of no difference in change over time across groups against the alternative hypothesis of a difference between the two arms. An additional term in the model was fitted to account for the repeated measures pre- and post-intervention. “Participant” was declared a random effect. An unstructured covariance pattern between baseline and post-intervention was used. The Kenward and Roger’s method for correcting for the fixed effects, standard error bias by inflation of the variance and Satterthwaite’s adjustment to the degrees of freedom has also been applied to cater for the small sample size. Analyses of endpoints with non-normal variance for analysis have been transformed to the log_10_ scale for analysis. Results were back transformed for interpretation and represent geometric means. Cohens *d* was calculated as a measure of effect size with 0.2, 0.5, and 0.8 considered small, medium and large effects in the CT compared to the AC group.^47^

#### Covariate analysis

An additional analysis was undertaken to investigate the effects of the following covariates on all outcome measures: age, education, levodopa equivalency daily dose LEDD;^48^ years since diagnosis, and the amount of days between CT completion and FU assessment (days until FU) on each outcome. Only significant results are reported.

### Data availability statement

The data that support the findings of this study are available from the corresponding author upon reasonable request.
